# Influence of Latitude on the Prevalence of Kawasaki Disease: A Retrospective Cohort Study from the Taiwan National Health Insurance Database and Review of the Literature

**DOI:** 10.3390/ijerph15050845

**Published:** 2018-04-25

**Authors:** Chaw-Liang Chang, Chih-Shung Wong, Yi-Chen Yang, Nan-Chang Chiu

**Affiliations:** 1 Department of Biological Science and Technology, National Chiao Tung University, Hsinchu 30068, Taiwan; juliancsr@yahoo.com.tw; 2 Department of Pediatrics, Cathay General Hospital, Hsinchu 30060, Taiwan; 3 Department of Anesthesiology, Cathay General Hospital, Taipei 10630, Taiwan; w82556@gmail.com; 4 School of Medicine, Fu-Jen Catholic University, New Taipei 24205, Taiwan; 5 Smart Healthcare Promotion Office, National Chiao Tung University, Hsinchu 30010, Taiwan; vanessaa5518@gmail.com; 6 Department of Pediatrics, MacKay Children’s Hospital, Taipei 10449, Taiwan; 7 Mackay Junior College of Medicine, Nursing and Management, New Taipei City 11260, Taiwan; 8 Mackay Medical College, New Taipei City 25245, Taiwan

**Keywords:** Kawasaki disease, latitude, climate, prevalence

## Abstract

*Background:* Countries at higher latitudes have higher incidence rates of Kawasaki disease (KD) than do countries at lower latitudes in the Asian and West Pacific area. However, the precise influence of latitude on KD incidence rates requires further clarification. *Methods:* We searched the Longitudinal Health Insurance Database 2005 to retrieve patients’ medical records from 1996 to 2009. The patients with KD were categorized as living in northern, middle, and southern Taiwan; the period prevalence of KD for each area was determined. Climate variables, including temperature, sunshine duration, precipitation, and relative humidity, were collected from the Taiwan Central Weather Bureau. The effect of latitude on the period KD prevalence and the correlation between climate variables and KD prevalence were calculated. *Results:* After patients without complete data excluded, a total of 61,830 children up to 10 years old were retrieved, from which 404 patients with KD were recognized. The period prevalence of KD increased significantly with latitude (*p* = 0.0004). Climate variables associated with high temperature demonstrated a connection with KD prevalence; however, this correlation was not statistically significant. *Conclusions:* Our study demonstrated that higher latitude is associated with a higher KD prevalence in Taiwan.

## 1. Introduction

Kawasaki disease (KD) is an acute idiopathic systemic vasculitis characterized by fever, bilateral non-exudative conjunctivitis, mucositis, cervical lymphadenopathy, polymorphous eruption, and changes in the extremities [1,2]. Most patients with KD are younger than 5 years old, although the disease can also occur in adolescents [2]. KD is the leading cause of pediatric-acquired heart disease in North America, Europe, and Japan [2,3]. It results in coronary artery aneurysms in 20–25% of untreated cases. Coronary artery aneurysms may lead to myocardial infarction and sudden death [1].

Fifty years have passed since Tomisaku Kawasaki first reported an acute febrile mucocutaneous lymph node syndrome in 1967 [4]. Many etiologies of KD have been proposed, but the agent that triggers the inflammatory response remains unidentified [2]. Environmental factors have been proposed as triggering KD [5]. Evidence suggests that KD is likely caused by the final common pathway of many microbial infectious and environmental factors triggering inflammation in genetically susceptible individuals [2,6].

The incidence rates of KD differ among countries, however, males are predominantly affected [7,8]. The incidence rate of KD is higher in the Asian and West Pacific area [8]. Here, we observed that countries at higher latitudes have higher incidence rates of KD than do countries at lower latitudes; Japan (264.8/100,000) and Korea (194.7/100,000) have the highest and second-highest annual incidence rates [7,9]. The annual incidence rates of lower latitude countries, such as India (1.0–9.1/100,000) and Thailand (2.1–3.4/100,000), are much lower than those of higher latitude countries [10,11]. Whether this trend is contributed to by ethnic and genetic factors, geographic and environmental factors, or other unknown factors requires clarification.

With the Tropic of Cancer crossing its middle area, Taiwan, which has a 22°–25° north latitude, is located between the higher incidence rate and latitude countries (Japan and Korea) and lower incidence rate and latitude countries (India and Thailand). These special geographic characteristics provide a good model for testing the role of latitude in KD occurrence. In this study, through defining the period (1996–2009) prevalence of KD in northern, middle and southern Taiwan and by using the National Health Insurance Research Database (NHIRD), we evaluated the role of latitude on KD prevalence.

## 2. Methods

### 2.1. Database

The Taiwan NHIRD was launched in 1995. More than 98% of Taiwan’s population was enrolled in this program. Currently, approximately 25 million beneficiaries are registered in the NHIRD. The Longitudinal Health Insurance Database 2005 (LHID2005) contains data of 1,000,000 individuals randomly sampled from the 2005 NHIRD. No significant difference exists in the gender distribution, age distribution, and health care costs between the patients in the LHID2005 and the original NHIRD.

### 2.2. Standard Protocol Approvals and Patient Consents

This study was approved by the Institutional Review Board of the Cathay General Hospital (CGH-P104037). The protocol was reviewed and approved by the National Health Research Institute prior to data being released. Because the LHID2005 contains de-identified secondary data and information, no individual can be identified; the informed consent of subjects was therefore waived.

### 2.3. KD Period Prevalence

**Figure 1:**
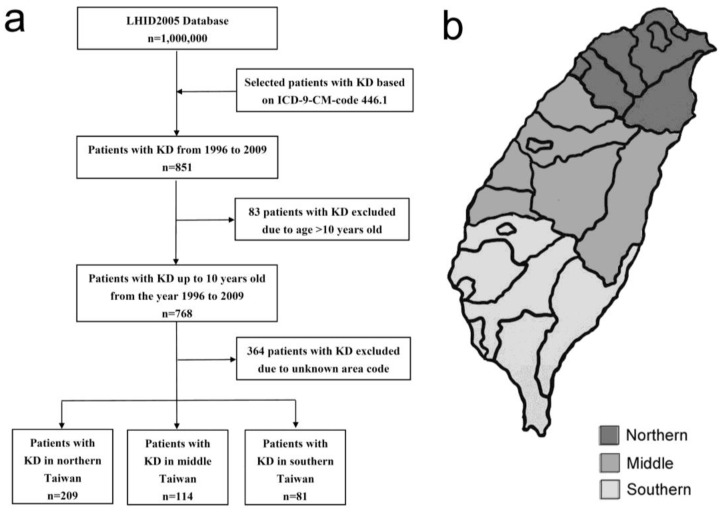
Flow diagrams of the study, (**a**) 404 Kawasaki disease patients up to 10 years old were recognized; (**b**) These patients were further categorized into 3 geographical regions: northern, middle, and southern Taiwan. KD: Kawasaki disease.

This was a retrospective cohort study. We searched the LHID2005 to retrieve patients’ medical records from 1996 to 2009. From this database, we selected outpatients based on the *International Classification of Diseases, Ninth Revision* (ICD-9) code for KD (446.1). Patients’ gender, date of diagnosis, age, and area code were retrieved. Patients up to 10 years old was selected and patients without complete data were excluded (Figure 1a). The gender distribution, age distribution, and period prevalence were calculated. According to the area code, the patients were further categorized into three geographical regions: northern, middle, and southern Taiwan (Figure 1b). The period (1996–2009) prevalence of KD in each area was determined and the effect of latitude on these values was analyzed.

### 2.4. Climate Variables

Climate variables of northern, middle and southern Taiwan, comprising the mean temperature, mean maximum temperature of each month, number of days with a mean temperature of ≥30 °C, number of days with a mean temperature of ≥25 °C, number of days with a mean temperature of ≤10 °C, mean minimum monthly temperature, sunshine duration, precipitation, and mean relative humidity, were collected from the Taiwan Central Weather Bureau (http://www.cwb.gov.tw/ eng/index.htm). The correlation between the climate variables and KD prevalence was calculated.

### 2.5. Statistical Analysis

SAS 9.1 for Windows (SAS Institute, Inc., Cary, NC, USA) was used for data retrieval and data analysis. Pearson’s chi-square test was applied for categorical data comparisons. Correlations were calculated using linear regression. The level of significance was set at *p* < 0.05 by using a 2-tailed comparison.

## 3. Results

After patients without complete data excluded, a total of 61,830 children up to 10 years old were retrieved from the LHID2005 during the study period of 1996–2009. Among them, 404 patients with KD were identified. Gender distributions and age percentages of patients with KD are listed in Table 1. The male–female ratio of patients with KD was 1.42 (237/167). Most KD cases occurred in children who were ≤5 years old (347/404; 85.9%) and approximately 60% (242/404; 59.9%) of KD cases occurred in children who were ≤2 years old (Table 1).

The distribution of KD among northern, middle, and southern Taiwan is listed in Table 2. More than half of patients with KD lived in northern Taiwan. The percentages of patients with KD in northern, middle, and southern Taiwan were 51.7% (209/404), 28.2% (114/404), and 20.1% (81/404), respectively. The period prevalence of KD from 1996 to 2009 was 65.3/10,000. The period prevalence of KD increased significantly (*p* = 0.0004) with latitude (46.5/10,000, 65.2/10,000, and 77.6/10,000 at southern, middle and northern Taiwan, respectively; Table 2). The differences of period prevalence between northern and southern Taiwan and between middle and southern Taiwan were also significant (*p* = 0.0001 and 0.0193, respectively). Northern Taiwan had a higher period prevalence than did middle Taiwan but no statistical difference existed between either (*p* = 0.1327).

The correlations between climate variables and KD prevalence are listed in Table 3. Among the climate variables, days with a mean temperature of ≥25 °C had the strongest connection with KD prevalence (*R*^2^ = 0.9837, *p* = 0.082). Mean temperature (*R*^2^ = 0.9264), mean maximum temperature of each month (*R*^2^= 0.8990), number of days with a mean temperature of ≥30 °C (*R*^2^ = 0.7364), and sunshine duration (*R*^2^ = 0.8185) also had a strong connection with KD prevalence. However, this connection was not statistically significant (Table 3).

## 4. Discussion

This study revealed that a higher latitude is associated with a higher period prevalence of KD in Taiwan; this is similar to a trend observed in Asia that countries and areas at higher latitudes have higher KD incidence rates (Table 4) [7,9,10,11,12,13,14,15,16,17,18,19,20,21,22,23,24,25], although the surveys of incidence rates for each country were not performed in the same year and the incidence rates of KD have been reported to increase with time [8]. Japan (latitude 24°–46°) has the highest KD incidence rate (264.8/100,000) in the world and Korea (latitude 33°–39°) has the second-highest annual KD incidence rate (194.7/100,000) [7,9]. Taiwan (latitude 22°–25°) has the third-highest KD incidence rate in the world [14]. The annual KD incidence rates of lower latitude countries, such as India (1.0–9.1/100,000) and Thailand (2.1–3.4/100,000), are much lower than those of higher latitude countries [10,11]. This association between latitude and KD incidence rate also exists in Canada (26.2/100,000) and the United States (19.0/100,000) [16,17]; however, no similar trend exists in Europe (Table 4). The low KD incidence and lack of association between latitude and KD incidence in Europe may indicate that different outbreaks of KD in different areas seem to be associated with different trigger factors and no single major trigger factor exists in Europe.

This association of latitude and KD incidence rate in Asia and North America may be partially explained by a hypothesis put forth by Rodó et al. [5]. By analyzing seasonal variations and epidemics of KD in Japan, Hawaii, and San Diego, they concluded that KD cases are often linked to large-scale wind currents originating in northeastern China and traversing the north Pacific, suggesting that an airborne trigger is carried in the troposphere [5,26]. Because the higher latitude countries in the Asia and West Pacific area, such as Japan and Korea, are closer to northeastern China, which is where the airborne trigger possibly originates from, genetically vulnerable individuals in those countries are more likely to be influenced by the unknown airborne trigger.

In addition to the hypothesis by Rodó et al., the association of latitude and KD incidence may be explained by genetic predisposition, environmental factors, climate variables, infection or post-infectious inflammatory response. Like many inflammatory disorders, such as inflammatory bowel disease and vasculitis, KD is believed to be associated with exposure to seasonal infectious and environmental factors in genetically susceptible individuals during the maturation of the immune system [2,6]. Certain external factors activate the cascade of processes involving proinflammatory cells that finally leads to diseases such as KD and vasculitis [27].

The hypothesis that environmental factors trigger the onset of inflammatory disorders has been proposed [5,26,28,29,30]. A study in Central Chile reported an association of KD with dust transported from the Atacama Desert; it determined that meteorological variables can explain 38% of variances in KD incidence rates [28]. Although the environmental triggers of KD remain unknown, several have been studied. *Candida* species have been deemed as possible triggers of KD because they have been observed as the dominant fungal species in aerosols transported by wind [26]. This finding is compatible with the KD mouse model of coronary arteritis. Intraperitoneal injections of *Candida albicans*-derived substances have long been used to create coronary arteritis mimicking KD in mice [31]. Exposure to O_3_ has also been demonstrated to increase the risk of KD in children [32]; however, short-term exposure to air pollution and fine particulate matter (diameter  ≤  2.5 µm) as well as residential proximity to water have failed to be associated with KD [29,30,33].

In this study, climate variables associated with high temperature, including the mean temperature, days with a mean temperature of ≥25 °C, mean maximum temperature of each month, number of days with a mean temperature of ≥30 °C, and sunshine duration, demonstrated a connection with KD prevalence; however, this correlation was not significant (Table 3). Although not significantly so, a high mean temperature and more sunshine exposure were connected to lower KD prevalence rates. Similar results were also reported by Abrams et al., they found that the KD incidence was negatively associated with the ambient temperature, whereas in contrast, KD incidence was positively associated with precipitation [34]. Our study also demonstrated a similar result between KD prevalence and precipitation; however, the association was not statistically significant (Table 3). Additionally, a study in Shanghai revealed that short-term exposure to high temperatures may significantly increase the incidence rate of KD [33]. The contradictory results about the effects of long-term and short-term exposure to high temperatures on KD rates reflect a complexity of KD etiologies; KD may be caused by a combination of microbial infection and environmental triggers.

The latitude and climate may influence the prevalence of KD in several ways. Firstly, the latitude and climate may affect the abundance of vectors and blooming, transmissibility and survival of specific pathogens (viruses, bacteria or others), which may trigger the inflammatory process in genetically susceptible individuals. Secondly, the latitude may affect the wind current which brings airborne triggers, such as dust, air pollutants, and fungal spore. Thirdly, climate and temperature may also influence lifestyle and human behavior (e.g., people may stay longer time indoor and exercise less in cold or wet weather), which may lead to different KD prevalence [34].

In addition to the environmental factors and climate variables, several etiologies of KD have been proposed. Based on the seasonal clustering of cases and the similarity of KD to other pediatric febrile exanthems, an infectious etiology is suspected [2]. Many bacteria, bacterial toxins, and viruses have been proposed to cause KD; however, no definitive causative pathogens have been identified [2,35]. Another risk factor of KD is genetic susceptibility. In the United States, the incidence rates of KD are particularly high among children of Asian and Pacific Islander descent [2]. Additionally, epidemiological data from Hawaii and California suggest that the differences in incidence rates of KD among ethnicities are related to genetic factors [36,37]. Higher rates of KD in the siblings of index cases and twins also suggest that genetic predisposition is a risk factor for KD [1,8]. However, no single genetic marker associated with KD can account for even 1% of disease susceptibility [2].

This study used data from the LHID2005, which contains data of 1,000,000 randomly sampled individuals; it offers an accurate depiction of prevalence and epidemiological features of KD. However, this study still has its limitations. Because the LHID2005 contains de-identified secondary data, we are not able to contact individuals to complete any missing data, leading to individuals being excluded. The area distribution in this study (51.7%, 28.2%, and 20.0% in northern, middle, and southern Taiwan) is similar to that of previous study (42%, 27%, and 28% in northern, middle, and southern Taiwan) [38], but the exclusion of 47% (364/768) of all KD cases, due to missing data, might skew the result. Additionally, the LHID2005 contains no details of environmental factors such as temperature, humidity, wind, atmospheric particle counts, and pollutants. Cross analyzing LHID2005 with other environmental databases or a large-scale longitudinal nationwide survey may further clarify the real trigger factors. Considering this study is based on Taiwan LHID 2005 and the association between latitude and KD is observed mainly in the Asian and West Pacific area, further study is necessary to determine whether the association between the latitude and KD could be applied to other regions of the world.

## 5. Conclusions

Our study suggested that higher latitudes are associated with higher prevalence rates of KD in Taiwan and provided evidence that environmental factors may trigger KD inflammatory processes. Latitude and climate scale should be considered in future KD studies.

## Figures and Tables

**Table 1:** Characteristics of patients with Kawasaki disease in Taiwan (1996–2009).

Characteristics		Population ≤ 10 years		Kawasaki Disease	
		61,830		404	
Gender	Male	32,064	51.9%	237	58.7%
	Female	29,766	48.1%	167	41.3%
Age, year	<1	4087	6.6%	35	8.7%
	1	4476	7.2%	121	30.0%
	2	4847	7.8%	86	21.3%
	3	5009	8.1%	47	11.6%
	4	5257	8.5%	37	9.2%
	5	6386	10.3%	21	5.2%
	6	5930	9.6%	18	4.5%
	7	5636	9.1%	15	3.7%
	8	6829	11.0%	12	3.0%
	9	6765	10.9%	6	1.5%
	10	6608	10.7%	6	1.5%

**Table 2:** Prevalence of Kawasaki disease in Northern, Middle, and Southern Taiwan.

Region	Population ≤ 10 years	Kawasaki Disease	Prevalence	*p-*Value
Northern (N)	26,933	209 (51.7%)	77.6/10,000	0.0004
Middle (M)	17,486	114 (28.2%)	65.2/10,000	
Southern (S)	17,411	81 (20.0%)	46.5/10,000	
N vs. M				0.1327
N vs. S				0.0001
M vs. S				0.0193
Total	61,830	404	65.3/10,000	

**Table 3:** Climate variables and Kawasaki disease prevalence.

Area	Mean Temperature (°C)	Mean Monthly Maximum Temperature (°C)	Maximun Temperature ≥ 30 °C (d/y)	Mean Temperature ≥ 25 °C (d/y)	Mean Temperature ≤ 10 °C (d/y)	Mean Monthly Minimum Temperature (°C)	Sunshine Duration (hr/y)	Precipitation (mm/y)	Mean Relative Humidity (%)
Northern (Taipei)	23.0	26.6	134.0	153.7	7.4	20.4	1405.2	2405.1	76.6
Middle (Taichung)	23.3	28.1	162.2	171.3	10.7	19.8	2043.2	1773	75.6
Southern (Kaohsiung)	25.1	28.8	165.7	215.7	0.9	22.1	2212.2	1884.9	75.9
Linear regression	Prevalence = b0 + b1 × Climate								
*R* ^2^	0.9264	0.8990	0.7364	0.9837	0.5409	0.6228	0.8185	0.4787	0.3510
*t*-Value	−3.548	−2.984	−1.671	−7.761	1.085	−1.285	−2.123	0.958	0.735
*p*-Value	0.175	0.206	0.343	0.082	0.474	0.421	0.280	0.514	0.596

Note: d/y: average total days per year; hr/y: average total hours per year; mm/y: millimeter per year.

**Table 4:** Incidence rates of Kawasaki disease in Asia, North America, and Europe.

Region	Latitude	Incidence ^a^	Period	Citation
Asia				
Japan	24–46	264.8	2012	Makino et al. 2015 [7]
Korea	33–39	194.7	2014	Kim et al. 2017 [9]
Beijing	39	55.1	2004	Du et al. 2007 [12]
Shanghai	31	55.5	2012	Chen et al. 2016 [13]
Taiwan	22–25	69.0	2003–2006	Huang et al. 2009 [14]
Hong Kong	22	39.0	1994–2000	Ng et al. 2005 [15]
India	8–37	7.0	2014	Singh et al. 2016 [10]
Thailand	5–20	2.6	2002	Durongpisitkul et al. 2006 [11]
North America				
Canada	41–83	26.2	1995–2006	Lin et al. 2010 [16]
United States	18–71	20.8	2006	Holman et al. 2010 [17]
Europe				
Finland	59–70	11.4	1998–2009	Salo et al. 2012 [18]
Norway	57–80	5.4	1998–2009	Salo et al. 2012 [18]
Sweden	55–69	7.4	1998–2009	Salo et al. 2012 [18]
Ireland	51–55	15.2	1996–2000	Lynch et al. 2003 [19]
Netherlands	50–53	5.8	2008–2012	Tacke et al. 2014 [20]
England	49–60	8.4	1998–2003	Harnden et al. 2009 [21]
Germany	47–54	7.2	-	Jakob et al. 2016 [22]
France	42–51	9.0	2005–2006	Heuclin et al. 2009 [23]
Portugal	37–42	6.5	2000–2011	Pinto et al. 2017 [24]
Italy	35–47	14.7	2008–2013	Cimaz et al. 2017 [25]

^a^ Incidence rates are reported per 100,000 children of <5 years of age.
